# The Epidemiology of Depression and Diabetes Distress in Type 2 Diabetes in Kuwait

**DOI:** 10.1155/2020/7414050

**Published:** 2020-06-01

**Authors:** Ebaa Al-Ozairi, Abdulla Al Ozairi, Clare Blythe, Etab Taghadom, Khalida Ismail

**Affiliations:** ^1^Dasman Diabetes Institute, Kuwait; ^2^Faculty of Medicine, Kuwait University, Kuwait; ^3^Department of Psychiatry, Faulty of Medicine, Kuwait University, Kuwait; ^4^Department of Psychological Medicine, Institute of Psychiatry, Psychology and Neuroscience, King's College London, London, UK

## Abstract

This study is aimed at describing the prevalence of and risk factors for depression and diabetes distress in people with type 2 diabetes and whether depression and distress are independently associated with worse biomedical outcomes. The study was of cross-sectional design. The setting was the Dasman Diabetes Institute, Kuwait. The Patient Health Questionnaire-9 (PHQ-9) was used to measure the prevalence of depression, defined as a score ≥ 10 (depression caseness). The Problem Areas in Diabetes (PAID) was used to measure diabetes-related distress. Data on biomedical outcomes, lifestyle factors, and sociodemographic information were collected. The prevalence of depression and diabetes distress caseness was 29% and 14%, respectively. Depression caseness patients were more likely to be female (60%; *p* = 0.001), have Kuwaiti nationality (68%, *p* = 0.121), were on insulin (67%, *p* = 0.001), have higher body mass index (*p* = 0.047), were less physically active (78%; *p* = 0.034), have a higher PAID score (*p* < 0.001), and have hypertension (74%, *p* = 0.047). After adjustment of sociodemographics (age, gender, and marital status) and body mass index, the prevalence of depression was associated with higher HbA1c (*B* = 0.04, 95% confidence interval 0.01 to 0.60), while diabetes distress had a weak association with HbA1c (*B* = 0.13, 95% confidence interval 0.04 to 0.22). In conclusion, people with type 2 diabetes in Kuwait have a high prevalence of depression but lower diabetes distress and this was associated with worse glycaemic control.

## 1. Introduction

In predominantly Western settings, the prevalence of depression is increased around twofold in people with diabetes compared with the general population [1]. In people with diabetes, depression is associated with worse diabetes outcomes such as reduced diabetes self-care [2], worse glycaemic control [3], and increased premature mortality [4–6]. Interventions that focus on depression alone tend not to lead to improved glycaemic control, while interventions that integrate the management of both depression and type 2 diabetes (T2DM) have better outcomes [7].

Previous epidemiological studies and interventions on depression and diabetes-related psychological distress have mostly been based in Western settings and very little in Middle East Muslim populations. In this region, diabetes is a major public health problem with a prevalence of around 20% in Kuwait, Qatar, Saudi Arabia, and the United Arab Emirates [8]. The prevalence of diabetes is increasing more rapidly in Asia than elsewhere in the world; in India, it is predicted to rise to 134.3 million by 2045 [8]. The risk of depression appears to be 3-fold in people with T2DM in South Asian populations [9, 10]. In Japan, diabetes distress, but not depression, was associated with worse glycaemic control suggesting there may be cultural variations in the reporting of these two related psychological constructs [11]. Religious and cultural values in a predominantly Muslim affluent population and living in a hot climate effect on the lifestyle may be important contextual factors for depression, diabetes-related psychological distress, and the burden of diabetes self-management. The aim of this study is to describe the prevalence of depression and diabetes distress in a typical Arabic Muslim T2DM population and to test whether they are associated with worse glycaemic control adjusting for sociodemographic and lifestyle factors and other diabetes-related biomedical outcomes.

## 2. Materials and Method

### 2.1. Study Setting and Design

The study was conducted at the Dasman Diabetes Institute (DDI), which is a fully integrated clinical and research facility that serves the Kuwaiti population. The study was given ethical approval by the Institutional Review Boards of Dasman Diabetes Institute and the Ministry of Health. The study used a cross-sectional design.

### 2.2. Sample

The study sample was collected from the people with T2DM who attended the Diabetes Outpatient Department and Clinical Research Department. Individuals who met study eligibility criteria were invited to take part in this study. The participation was voluntary, and responses were anonymous and handled confidentially. The inclusion criteria included adults aged 21 years and older, who were residents and regularly attending clinics at DDI. The exclusion criteria were pregnancy, psychosis, dementia, non-Arabic and/or English speaking, and any religion other than Islam.

### 2.3. Measures

#### 2.3.1. Demographic and Lifestyle

Demographic variables included gender, age, nationality (either Kuwait native or non-Kuwaiti), occupation, marital status, and number of children. Participants were interviewed about their lifestyle, namely, smoking status, alcohol consumption, physical activity, and diet.

#### 2.3.2. Clinical Data

The participants' duration of T2DM was recorded. The body weight (kg), height (measured to closest 0.5 cm), and waist circumference (cm) were measured. The BMI was calculated as weight/height^2^ (kg/m^2^) and used as an overall index of adiposity. The values of HbA1c -Diabetes Control and Complications Trial (DCCT) which were standardised at the DDI laboratory, were further converted into mmol/mol values following the International Federation of Clinical Chemistry (IFCC) standard.Complete lipid profiles including total cholesterol, high-density lipid cholesterol (HDL-C, mmol/L), low-density lipid cholesterol (LDL-C, mmol/L), and triglycerides (TG, mmol/L) were measured. Dyslipidaemia was defined as having a total cholesterol greater than 4 mmol/L, an LDL of more than 2.0 mmol/L, or the use of cholesterol-lowering medication. Blood pressure was measured twice (in the semirecumbent position) over the brachial artery of the dominant arm using an automated sphygmomanometer. Patients were diagnosed as having hypertension if the systolic blood pressure (SBP) ≥ 130 mmHg and/or the diastolic blood pressure (DBP) ≥ 80 mmHg on two separate occasions and/or if the patients were on antihypertensive medication. Diabetic retinopathy was measured using digital retinal photographs completed and classified by a trained ophthalmologist. Diabetic nephropathy was measured using the albumin : creatinine ratio. Neuropathy was assessed by pinprick sensation, vibration perception (using a 128 Hz tuning fork), 10 g monofilament pressure sensation at the distal plantar aspect of both great toes and metatarsal joints, and assessment of ankle reflexes.

#### 2.3.3. Assessment of Depression

The Patient Health Questionnaire-9 (PHQ-9), which is comprised of a 9-question depression scale, was used to screen for the presence and severity of depressive symptoms according to the Diagnostic and Statistical Manual of Mental Disorders-IV criteria (DSM-IV), over the past 2 weeks. Each of the nine symptoms was scored as “0” (not at all), “1” (several days), “2” (more than half the days), or “3” (nearly every day) giving a range of scores between 0 and 27; the higher the score, the greater the severity [12]. A PHQ-9 score ≥ 10 was defined as positive for depression caseness, which has satisfactory validity in individuals with diabetes. The Arabic translation of PHQ-9 has been validated and is reliable [13]. The history of depression was assessed by asking patients whether they have had a previous diagnosis of depression or if they have received care by a psychiatrist or psychologist in the past [14].

#### 2.3.4. The Problem Areas in Diabetes Scale (PAID)

The PAID is a measure of a person's emotional adjustment in response to living with diabetes [15]. Each of the 20 questions corresponds to a potential problem of living with diabetes (e.g., “feeling constantly concerned about food and eating”) and is rated as “0” (not a problem), “1” (minor problem), “2” (moderate problem), “3” (somewhat serious problem), and “4” (serious problem). The final PAID score is calculated by summing-up all the 20-item scores and multiplying by 1.25. A minimum score of 0 indicates no diabetes-related distress, whereas a maximum score of 100 indicates significant diabetes-related distress [16]. A score of more than 40 is considered clinically significant psychological distress [17].

#### 2.3.5. Statistical Analysis

Data were analysed using SPSS statistical software (version 25.0, IBM SPSS Statistics; IBM Corp., Armonk, NY). The study characteristics were described using the mean (standard deviation) or proportions (%). For the association statistical tests, we analysed the nested sample group of patients who had completed both PHQ-9 and PAID questionnaires. The association of depression caseness or PAID with study characteristics was conducted using *t*-tests or regression analysis for odds ratio for continuous and categorical explanatory variables, respectively. Multinomial linear regression was used to adjust for those lifestyle and biomedical variables with a statistically significant association with depression as potential confounders of the hypotheses.

## 3. Results

Of the original cohort of *n* = 893 participants, *n* = 465 had both PHQ9 and PAID scores. The demographic and clinical characteristics of original cohort are reported in supplementary table 1. The demographic and clinical characteristics of the nested sample of participants with both PHQ9 and PAID scores (*n* = 465) are reported in Table 1. In the nested sample, the mean age was 55.3 (SD 10.1) years and the mean duration of T2DM was 12.5 (8.2) years. The mean % HbA1c value was 8.5 (1.9) or 69.4 (20.5) mmol/mol. The proportion of males and females was almost similar (52% versus 48%); the overwhelming majority were married (91%) and of Kuwaiti nationality (73%). The majority of participants had dyslipidaemia (73%) and history of hypertension (67.5%). Around 1/3^rd^ of the participants had at least one microvascular complication, and most did not report a past history of depression (96%).

There were no statistical differences in the distribution of sociodemographic or biomedical variables between the original and nested cohort (Supplementary table 2).

In the nested sample, depression caseness patients also had a higher PAID score (mean (SD) 28.6 (25.7)) indicating higher levels of diabetes distress compared with those who were PHQ-9 case negative (Table 1). In the PAID analysis, a quarter of the sample had scored zero (*n* = 113) thus skewing the distribution to the left. The median PAID score was 10 (interquartile range 1.3-28.8). When PAID scale was stratified into high distress (scores ≥ 40) versus low distress (<40), high distress patients were more likely to be a little younger (by 2 years), more likely to be female, more likely to be Kuwaiti, and more likely to have a higher HbA1c and PHQ-9 score, but otherwise, there were no other sociodemographic or biomedical differences between the two categories (Table 1).

In model 1 of the multinomial regression using the PHQ-9 as a continuous variable, there was a significant effect of depression symptoms on glycaemic control (*B* = 0.03, *p* = 0.039), after adjusting for variables which had a significant association with PHQ-9 as potential confounders (namely, gender, BMI, prescribed insulin, having past history of depression and hypertension, and not following any physical activity programme (see Table 2). This represented that every one-point increase in PHQ-9 score HbA1c was associated with a 0.03 mmol/mol increase in the HbA1c. In model 2, the PAID diabetes distress measure was added to model 1 and the association between depression and glycaemic control disappeared (*B* = 0.01, *p* = 0.276). When using the PAID as a continuous variable in model 1, the size of association between diabetes distress and glycaemic control was high and statistically significant (*B* = 0.13, *p* = 0.008) when adjusting for potential confounders. The PAID score was not associated with the presence of hypertension, being on insulin treatment or physically active. When the depression measure was added, the association between diabetes distress and glycaemic control was smaller but remained statistically significant (*B* = 0.09, *p* = 0.044).

## 4. Discussion

In this study, the prevalence of depressive symptoms and diabetes-related distress was evaluated in a large sample of people with T2DM in Kuwait. We found a high prevalence of depression with nearly a third of patients being screened positive for depression using the accepted cut-off for the PHQ-9; this is higher than the often cited 25% pooled prevalence [18]. The mean level of diabetes-related distress as measured by the PAID was lower compared with findings in Western populations [19]. We found that depression was associated with worse glycaemic control and being on insulin treatment—in essence a poor prognostic group. On the other hand, diabetes distress had a strong association with glycaemic control and was not associated with other markers of poor diabetes control.

The strength of this study is that it is a relatively large sample of a Middle Eastern Muslim population with T2DM in Kuwait as the Dasman Diabetes Institute serves both the local population of Kuwait City and is a tertiary diabetes centre for Kuwait. A limitation is that this is a cross-sectional study, and therefore, causal inferences cannot be made. However, as it is one of the few studies to examine the prevalence of depression in people with T2DM in non-Western setting, these findings provide important information on the impact of depression in a different cultural setting. Although we did not identify a systematic bias, it is noteworthy that in regression analysis of the original cohort which had a larger sample for depression, the unadjusted associations between depression and baseline variables were similar to the nested sample.

While it is often reported that there are cultural differences leading to greater stigma associated with depression and the idioms used to express it in the Asian continent, our study found the opposite that Muslim Middle Eastern patients are willing to acknowledge depressive symptoms when completing self-report questionnaires. On the other hand, the lower levels of diabetes-related distress suggest that perhaps as a population, people with T2DM in the Middle East are less worried than their Western counterparts. This has been borne in qualitative studies suggesting that people with T2DM tend to perceive diabetes as less serious than cancer [20] and as it is becoming so prevalent, almost the norm.

The association between depression and glycaemic control and diabetes complications is well documented in the literature [3, 21], and our findings support the notion that the impact of depression on diabetes is not necessarily culture bound [11]. Patients with diabetic retinopathy are reportedly more likely to express negative beliefs about their diabetes than those without retinopathy, after adjusting for age, gender, diabetes duration, and presence of other diabetic complications [22].

There have been fewer reports that depression is also associated with insulin treatment per se, and there are several possible reasons; depression has led to reduced self-care and adherence to oral medication; there may be residual confounding of progressive *β* cell destruction and/or associated excess inflammation contributing to the depressive symptomology; there may be reverse causation with insulin increasing the risk of depression. It is also possible that these patients whose glycaemic control is poor have more often hypoglycaemia, which has been shown to be associated with depressive symptoms.

People with depression were more often female, in keeping with earlier studies where depression has been found more prevalent in women with T2DM compared with the general population [23, 24]. Non-Kuwaiti nationality usually denotes lower social and economic status which may explain why this group was more likely to be depressed.

Patients with depression caseness had higher diabetes-related distress scores and worse glycaemic control. After adjusting for potential confounders and diabetes distress score, the relationship between depression and glycaemic control disappeared suggesting that either the predominant psychological problem is diabetes distress or there is a significant conceptual overlap between the two psychological constructs. The literature is divided in this respect with some studies finding a stronger association for depression than diabetes distress [25–27] on glycaemic control and others the reverse [11, 28]. In our study, the observation that PAID scores are highly correlated with depression suggests that the former were likely to be part of the depressive cognitive set [26]. We also noted a highly skewed distribution of distress with a quarter stating that they had no worries about living with their diabetes. This raises the question whether diabetes distress is more culturally dependent than depression. This remains an important area of study as it informs which psychological processes should be targeted for intervention.

## 5. Conclusions

In conclusion, this cross-sectional study showed that patients with T2DM in Kuwait have a high prevalence of depression and this was associated with worse diabetes outcomes and that insulin therapy appears to confound the association between depression and glycaemic control.

## Figures and Tables

**Table 1:** Distribution of explanatory variables stratified by PHQ-9 depression status and PAID score for diabetes distress in a nested sample (*n* = 465).

Explanatory variable		Total sample (*n* = 465) Mean (SD)/proportion (%)	PHQ-9 case positive^∗^ (*n* = 136) Mean (SD)/proportion (%)	PHQ-9 case negative^∗∗^ (*n* = 329) Mean (SD)/proportion (%)	Mean difference or odds ratio [95% confidence interval] (*p* value)^†^	PAID ≥ 40^∗∗∗^ (*n* = 69) Mean (SD)/proportion (%)	PAID < 40 (*n* = 396) Mean (SD)/proportion (%)	Mean difference or odds ratio [95% confidence interval] (*p* value)^†^
PHQ-9 score (range 0-27)		7.0 (5.3)	13.7 (3.5)	4.2 (2.8)	9.53 [8.92, 10.13] (<0.001)	11.2 (5.3)	6.2 (4.9)	5.0 [3.72, 6.28] (<0.001)
Age (year)		55.3 (10.1)	54.5 (11.3)	55.6 (9.6)	-1.14 [-3.17, 0.88] (0.268)	53.3 (10.5)	55.7 (10.0)	-2.31 [-4.9, 0.28] (0.080)
Duration T2DM (year)		12.5 (8.2)	13.1 (8.1)	12.3 (8.3)	0.79 [-0.89, 2.47] (0.356)	12.8 (8.1)	12.5 (8.3)	0.30 [-1.85, 2.46] (0.784)
Sex	Male	241 (51.8)	54 (39.7)	187 (56.8)	1	24 (34.8)	217 (54.8)	1
	Female	224 (48.2)	82 (60.3)	142 (43.2)	2.0 [1.3, 3.0] (0.001)	45 (65.2)	179 (45.2)	2.27 [1.33, 3.87] (0.003)
Nationality	Kuwaiti	341 (73.3)	93 (68.4)	248 (75.4)	1	60 (87.0)	281 (71.0)	2.7 [1.3, 5.68] (0.007)
	Non-Kuwaiti	124 (26.7)	43 (31.6)	81 (24.6)	1.4 [0.91, 2.2] (0.122)	9 (13.0)	115 (29.0)	1
Marital status	Married	411 (91.1)	116 (87.2)	295 (92.8)	1	61 (89.7)	350 (91.4)	1
	Separated/single	40 (8.9)	17 (12.8)	23 (7.2)	1.9 [0.97, 3.65] (0.062)	7 (10.3)	33 (8.6)	1.21 [0.51, 2.87] (0.654)
Employment status	Employed	208 (51.6)	61 (51.3)	147 (51.8)	1	25 (42.4)	183 (53.2)	1
	Unemployed	195 (48.4)	58 (48.7)	137 (48.2)	1.02 [0.66, 1.57] (0.927)	34 (57.6)	161 (46.8)	1.5 [0.88, 2.70] (0.126)
Past history of depression	Yes	17 (3.7)	11 (8.1)	6 (1.8)	4.7 [1.71, 13.08] (0.003)	5 (7.2)	12 (3.0)	2.5 [0.85, 7.33] (0.095)
	No	448 (96.3)	125 (91.9)	323 (98.2)	1	64 (92.8)	384 (97.0)	1
HbA1c (mmol/mol)		69.4 (20.5)	71.8 (20.1)	68.5 (20.6)	3.31 [-0.77, 7.40] (0.112)	74.9 (20.6)	68.5 (20.3)	6.39 [1.17, 11.61] (0.016)
BMI (kg/m^2^)		33.6 (6.8)	34.3 (6.6)	32.9 (7.0)	1.36 [0.01, 2.69] (0.047)	34.6 (5.7)	33.0 (7.0)	1.89 [0.17, 3.60] (0.031)
Serum triglycerides (mmol/L)		1.8 (1.0)	1.8 (1.0)	1.7 (1.1)	0.07 [-0.13, 0.27] (0.499)	1.9 (0.9)	1.7 (1.1)	0.11 [-0.15, 0.37] (0.402)
Serum total cholesterol (mmol/L)		4.6 (1.1)	4.7 (1.0)	4.6 (1.1)	0.14 [-0.77, 0.35] (0.208)	4.7 (0.9)	4.6 (1.1)	0.14 [-0.13, 0.41] (0.311)
Serum HDL (mmol/L)		1.1 (0.4)	1.2 (0.5)	1.1 (0.4)	0.06 [-0.23, 0.15] (0.149)	1.2 (0.5)	1.1 (0.4)	0.07 [-0.04, 0.17] (0.247)
Serum LDL (mmol/L)		2.7 (0.9)	2.7 (0.9)	2.7 (1.0)	0.01 [-0.18, 1.96] (0.955)	2.7 (0.8)	2.7 (1.0)	-0.02 [-0.26, 0.22] (0.874)
Blood pressure (mmHg)	Systolic	130 (17.7)	131.8 (18.9)	129.8 (17.1)	1.97 [-1.58, 5.53] (0.277)	135 (17.8)	129.6 (17.5)	5.49 [0.92, 10.05] (0.019)
	Diastolic	74 (10.9)	74.7 (9.9)	73.8 (11.3)	0.85 [-1.34, 3.04] (0.446)	75.1 (11.2)	73.9 (10.8)	1.22 [-1.60, 4.04] (0.396)
Diagnosis of dyslipidaemia	Diagnosis	339 (72.9)	99 (72.8)	240 (72.9)	1.0 [0.63, 1.56] (0.973)	52 (75.4)	287 (72.5)	1.2 [0.64, 2.10] (0.619)
	None	126 (27.1)	37 (27.2)	89 (27.1)	1	17 (24.6)	109 (27.5)	1
Retinopathy	Diagnosis	132 (28.4)	42 (30.9)	90 (27.4)	1.8 [0.76, 1.84] (0.443)	17 (24.6)	115 (29.0)	0.8 [0.44, 1.44] (0.455)
	None	333 (71.6)	94 (69.1)	239 (72.6)	1	52 (75.4)	281 (71.0)	1
Nephropathy	Diagnosis	93 (20.0)	28 (20.6)	65 (19.8)	1.05 [0.64, 1.73] (0.838)	14 (20.3)	79 (19.9)	1.0 [0.54, 1.93] (0.948)
	None	372 (80.0)	108 (79.4)	264 (80.2)	1	55 (79.7)	317 (80.1)	1
Neuropathy	Diagnosis	199 (43.3)	66 (48.9)	133 (40.9)	1.38 [0.92, 2.06] (0.117)	34 (49.3)	165 (42.2)	1.33 [0.80, 2.22] (0.275)
	None	261 (56.7)	69 (51.1)	192 (59.1)	1	35 (50.7)	226 (57.8)	1
Stroke	Yes	9 (1.9)	3 (2.2)	6 (1.8)	1.21 [0.29, 4.92] (0.786)	1 (1.4)	8 (2.0)	0.7 [0.09, 5.79] (0.752)
	No	456 (98.1)	133 (97.8)	323 (98.2)	1	68 (98.6)	388 (98.0)	1
Hypertension	Yes	314 (67.5)	101 (74.3)	213 (64.7)	1.57 [1.0, 2.45] (0.047)	49 (71.0)	265 (66.9)	1.2 [0.69, 2.12] (0.503)
	No	151 (32.5)	35 (25.7)	116 (35.3)	1	20 (29.0)	131 (33.1)	1
CVD	Yes	49 (10.5)	17 (12.5)	32 (9.7)	1.32 [0.70, 2.47] (0.377)	5 (7.2)	44 (11.1)	0.6 [0.24, 1.63] (0.339)
	No	416 (89.5)	119 (87.5)	297 (90.3)	1	64 (92.8)	352 (88.9)	1
Diet treated alone	Yes	37 (8.0)	12 (8.8)	25 (7.6)	1.17 [0.57, 2.41] (0.657)	6 (8.7)	31 (7.8)	1.10 [0.45, 2.80] (0.806)
	No	428 (92.0)	124 (91.2)	304 (92.4)	1	63 (91.3)	365 (92.2)	1
Oral agents alone	Yes	399 (86.2)	114 (83.8)	285 (87.2)	0.76 [0.43, 1.33] (0.345)	57 (83.8)	342 (86.6)	0.8 [0.40, 1.63] (0.543)
	No	64 (13.8)	22 (16.2)	42 (12.8)	1	11 (16.2)	53 (13.4)	1
Insulin	Yes	252 (54.4)	91 (66.9)	161 (49.2)	2.08 [1.37, 3.16] (0.001)	41 (60.3)	211 (53.4)	1.3 [0.78, 2.24] (0.294)
	No	211 (45.6)	45 (33.1)	166 (50.8)	1	27 (39.7)	184 (46.6)	1
Smoking	Smoker	98 (21.7)	25 (18.7)	73 (23.0)	1.3 [0.80, 2.16] (0.312)	12 (18.2)	86 (22.3)	0.8 [0.40, 1.51] (0.456)
	Ex/nonsmoker	354 (78.3)	109 (81.3)	245 (77.0)	1	54 (81.8)	300 (77.7)	1
Exercise	Plan	135 (29.0)	30 (22.1)	105 (31.9)	1	19 (27.5)	116 (29.3)	1
	None	330 (71.0)	106 (77.9)	224 (68.1)	1.65 [1.03, 2.64] (0.034)	50 (72.5)	280 (70.7)	1.09 [0.61, 1.92] (0.767)
PAID (range 0-92.5)		17.9 (20.1)	28.6 (25.7)	13.4 (15.2)	15.21 [11.42, 18.99] (<0.001)	56.8 (11.1)	11.1 (11.4)	45.66 [42.63, 48.69] (<0.001)

PHQ-9: Patients Health Questionnaire Nine; PAID: Problem Area in Diabetes; HDL: high-density lipoprotein; LDL: low-density lipoprotein; CVD: cardiovascular disease; BMI: body mass index. ^∗^PHQ − 9 score ≥ 10 was used to define PHQ-9 positive; ^∗∗^PHQ − 9 score < 10 was used to define PHQ-9 negative; ^∗∗∗^PAID score ≥ 40 is considered clinically significant psychological distress. ^†^Test statistic represents *t*-tests or regression analysis for odd ratio.

**Table 2:** Regression analysis of PHQ-9 or PAID continuous measure.

Independent variables	Depended variable			
	PHQ-9		PAID	
	Model 1	Model 2^∗^	Model 1	Model 2^∗∗^
	*B* (95% CI), *p* value	*B* (95% CI), *p* value	*B* (95% CI), *p* value	*B* (95% CI), *p* value
Gender	1.32 (0.33, 2.30), 0.009	0.95 (0.04, 1.86), 0.040	3.59 (-0.20, 7.38), 0.063	1.59 (-1.92, 5.11), 0.374
Past history of depression	4.47 (1.97, 6.97), <0.001	3.39 (1.08, 5.71), 0.004	10.5 (0.9, 20.19), 0.032	3.77 (5.23, 12.76), 0.411
Hypertension	0.68 (0.34, 1.70), 0.191	0.68 (0.26, 1.62), 0.155	0.01 (-3.92, 3.93), 0.997	1.04 (-2.58, 4.66), 0.573
Insulin user	0.94 (-0.10, 1.99), 0.078	0.96 (-0.008, 1.92), 0.052	-0.2 (-4.18, 3.88), 0.942	-1.58 (-5.30, 2.15), 0.406
Exercise	0.93 (-0.13, 2.0), 0.087	0.74 (-0.24, 1.73), 0.138	1.8 (-2.29, 5.94), 0.384	0.41 (-3.39, 4.21), 0.832
BMI	0.01 (-0.07, 0.086), 0.793	0.02 (-0.05, 0.09), 0.611	-0.08 (-0.37, 0.21), 0.603	-0.09 (-0.36, 0.18), 0.498
HbA1c	0.03 (0.001, 0.050), 0.039	0.01 (-0.01, 0.04), 0.276	0.13 (0.03, 0.22), 0.008	0.09 (0.01, 0.18), 0.044
Psychological measure	—	0.10 (0.08, 0.13), <0.001	—	1.52 (1.18, 1.85), <0.001

Model 1: regression included gender, past history of depression, hypertension, insulin user, exercise, body mass index (BMI), and glycaemic control (HbA1c). Model 2: included model 1 plus psychological measure. ^∗^Psychological measure was PAID. ^∗∗^Psychological measure was PHQ-9. PHQ-9: Patients Health Questionnaire Nine; PAID: Problem Area in Diabetes; BMI: body mass index.

## Data Availability

The participants' data used to support the findings of this study are restricted by the Ethical Review Committee of Dasman Diabetes Institute to protect patient privacy. Anonymous data are available from the corresponding author for researchers who meet the criteria for access to confidential data.

## Supplementary Materials

Supplementary Materials Supplementary Table 1: distribution of explanatory variables stratified by PHQ-9 depression. Supplementary Table 2: distribution of sociodemographic or biomedical variables in an original and nested cohort study.
