# Supplementary material for: The Epidemiology of Depression and Diabetes Distress in Type 2 Diabetes in Kuwait
Source: J Diabetes Res. 2020 Jun 1;2020:7414050. doi: 10.1155/2020/7414050 (PMC7288194; doi:10.1155/2020/7414050)
Supplement: Supplementary Materials — Supplementary Table 1: distribution of explanatory variables stratified by PHQ-9 depression. Supplementary Table 2: distribution of sociodemographic or biomedical variables in an original and nested cohort study. [file 7414050.f1.pdf]

Supplementary Table 1: Distribution of explanatory variables stratified by PHQ-9 depression.

Explanatory variable		Total cohort (n=893) Mean (SD)/ Proportion (%)	PHQ-9 case positive* (n=273) Mean (SD)/ Proportion (%)	PHQ-9 case negative** (n=620) Mean (SD)/ Proportion (%)	Mean difference, odds ratio [95% confidence interval] (p-value)†
PHQ-9 score		7.3 (5.4)	14.0 (3.7)	4.4 (2.7)	9.62 [9.2, 10.1] (<0.001)
Age (years)		55.3 (10.7)	54.3 (10.9)	55.6 (10.7)	-1.36 [-3.0, 0.1] (0.067)
Duration T2DM (years)		12.4 (8.5)	12.9 (8.4)	12.2 (8.5)	0.71 [-0.5, 1.9] (0.260)
Sex	Male	471 (52.7)	113 (41.4)	358 (57.7)	1
	Female	422 (47.3)	160 (58.6)	262 (42.3)	1.94 [1.5, 2.58] (<0.001)
Nationality	Kuwaiti	635 (71.1)	178 (65.2)	457 (73.7)	1
	Non-Kuwaiti	258 (28.9)	95 (34.8)	163 (26.3)	1.49 [1.1, 2.03] (0.010)
Marital status	Married	768 (90.1)	229 (88.8)	539 (90.7)	1
	Separated/ Single	84 (9.9)	29 (11.2)	55 (9.3)	1.24 [0.8, 2.0] (0.374)
Employment status	Employed	361 (50.3)	105 (48.6)	256 (51.0)	1
	Unemployed	357 (49.7)	111 (51.4)	246 (49.0)	1.10 [0.8, 1.51] (0.558)
Past history of depression	Yes	23 (2.6)	13 (4.8)	10 (1.6)	3.06 [1.32, 7.07] (0.009)
	No	869 (97.4)	259 (95.2)	610 (98.4)	1
HbA1c (mmol/mol)		70.0 (21.1)	72.2 (20.2)	69.1 (21.5)	3.05 [0.01, 6.1] (0.048)
BMI (Kg/m^2)		33.2 (6.8)	34.5 (6.8)	32.6 (6.7)	1.88 [0.9, 2.9] (<0.001)
Serum triglycerides (mmol/L)		1.8 (1.3)	1.9 (2.0)	1.8 (1.4)	0.13 [-0.1, 0.2] (0.262)
Serum total cholesterol (mmol/L)		4.6 (1.1)	4.7 (1.1)	4.6 (1.1)	0.09 [-0.1, 0.2] (0.294)
Serum HDL (mmol/L)		1.1 (0.5)	1.1 (0.5)	1.1 (0.5)	0.02 [-0.1, 0.1] (0.617)
Serum LDL (mmol/L)		2.7 (1.0)	2.8 (1.0)	2.7 (1.0)	0.06 [-0.1, 0.2] (0.457)
Blood pressure	Systolic	132 (17.6)	130.6 (18.5)	131.7 (16.8)	-1.11 [-0.15, 0.19] (0.372)
	Diastolic	75 (11)	75.3 (10.3)	74.6 (11.3)	0.72 [0.04, 1.0] (0.405)
Dyslipidaemia	Diagnosis	609 (68.4)	187 (68.8)	422 (68.2)	1.02 [0.75, 1.39] (0.865)
	None	282 (31.6)	85 (31.3)	197 (31.8)	1
Retinopathy	Diagnosis	257 (28.8)	91 (33.3)	166 (26.8)	1.37 [1.0, 1.86] (0.046)
	None	636 (71.2)	182 (66.7)	454 (73.2)	1
Nephropathy	Diagnosis	195 (21.8)	66 (24.2)	129 (20.8)	1.21 [0.86, 1.70] (0.262)
	None	698 (78.2)	207 (75.8)	491 (79.2)	1
Neuropathy	Diagnosis	352 (39.8)	117 (43.2)	235 (38.3)	1.22 [0.91, 1.63]

					(0.176)
	None	532 (60.2)	154 (56.8)	378 (61.7)	1
Stroke	Yes	14 (1.6)	5 (1.8)	9 (1.5)	1.27 [0.42, 3.81] (0.674)
	No	879 (98.4)	268 (98.2)	611 (98.5)	1
Hypertension	Yes	569 (63.7)	183 (67.0)	386 (62.3)	1.23 [0.91, 1.66] (0.172)
	No	324 (36.3)	90 (33.0)	234 (37.7)	1
CVD	Yes	98 (11)	33 (12.1)	65 (10.5)	1.17 [0.75, 1.83] (0.480)
	No	795 (89)	240 (87.9)	555 (89.5)	1
**Treatments**					
Diet	Yes	67 (7.5)	23 (8.4)	44 (7.1)	1.20 [0.71, 2.04] (0.488)
	No	826 (92.5)	250 (91.6)	576 (92.9)	**1**
Insulin	Yes	479 (53.8)	172 (63)	307 (49.7)	1.72 [1.29, 2.31] (<0.001)
	No	412 (46.2)	101 (37)	311 (50.3)	1
Oral Agents alone	Yes	756 (84.8)	228 (83.5)	528 (85.4)	0.86 [0.58, 1.27] (0.461)
	No	135 (15.2)	45 (16.5)	90 (14.6)	1
**Lifestyles**					
Smoking	Smoker	188 (21.7)	59 (22.3)	129 (21.4)	1.06 [0.74, 1.50] (0.744)
	Ex/ Non-Smoker	680 (78.3)	205 (77.7)	475 (78.6)	1
**Exercise**	Plan	272 (30.5)	66 (24.2)	206 (33.2)	1
	None	621 (69.5)	207 (75.8)	414 (66.8)	1.56 [1.12, 2.15] (0.007)
**Measures**					
**PAID**	Range: 0-92.5	17.7 (20.1)	28.9 (25.4)	13.2 (15.1)	15.87[12.2, 19.6] (<0.001)

PHQ-9, Patients Health Questionnaire Nine; **PAID**, Problem Area in Diabetes; **HDL,** High density lipoprotein; **LDL,** low density lipoprotein; **CVD,** Cardiovascular disease, BM1, Body Mass Index.
*PHQ-9 score ≥10 was used to define PHQ-9 positive; **PHQ-9 score <10 was used to define PHQ-9 negative.
†Test statistic represents t-tests for continuous or odd ratio from the regression analysis.

Supplementary Table 2: Distribution of sociodemographic or biomedical variables in original and nested cohort study.

Variables		Cohort sample (n= 893)<br>Mean (SD)/<br>Proportionate (%)	Nested sample (n= 465)<br>Mean (SD)/<br>Proportionate (%)	Mean Difference or Chi-test (p-value)*
PHQ9 score		7.3 (5.4)	7.0 (5.3)	0.34 (0.272)
PHQ9	Positive	273 (30.6)	136 (29.2)	0.25 (0.614)
	Negative	620 (69.4)	329 (70.8)	
Age	Years	55.2 (10.8)	55.3 (10.1)	0.11 (0.851)
Duration T2DM	Years	12.4 (8.5)	12.5 (8.2)	0.15 (0.754)
Gender	Male	471 (52.7)	241 (51.8)	0.10 (0.749)
	Female	422 (47.3)	224 (48.2)	
Nationality	Kuwaiti	635 (71.1)	341 (73.3)	0.75 (0.387)
	Non-Kuwaiti	258 (28.9)	124 (26.7)	
Marital Status	Married	768 (90.1)	411 (91.1)	0.33 (0.562)
	Single/separated	84 (9.9)	40 (8.9)	
Employment status	Employed	361 (50.3)	195 (48.4)	0.18 (0.668)
	Unemployed	357 (49.7)	208 (51.6)	
Past history of depression	Yes	23 (2.6)	17 (3.7)	1.24 (0.265)
	No	869 (97.4)	448 (96.3)	
BMI (kg/m2)		33.2 (6.8)	33.3 (6.5)	0.17 (0.670)
HbA1c (mmol/mol)		70.0 (21.1)	69.4 (20.5)	0.59 (0.622)
Serum triglycerides (mmol/L)		1.83 (1.6)	1.75 (1.0)	0.08 (0.369)
serum total cholesterol (mmol/L)		4.61 (1.1)	4.60 (1.1)	0.01 (0.908)
Serum HDL (mmol/L)		1.13 (0.5)	1.11 (0.4)	0.02 (0.514)
Serum LDL (mmol/L)		2.73 (1.0)	2.73 (0.9)	0.001 (0.991)
Dyslipidemia	Diagnosis	609 (68.4)	339 (72.9)	3.01 (0.083)
	None	282 (31.6)	126 (27.1)	
Retinopathy	Diagnosis	257 (28.8)	132 (28.4)	0.02 (0.879)
	None	636 (71.2)	333 (71.6)	
Neuropathy	Diagnosis	195 (21.8)	93 (20.0)	0.61 (0.432)
	None	698 (78.2)	372 (80.0)	
Neuropathy	Diagnosis	352 (39.8)	199 (43.3)	1.48 (0.224)
	None	532 (60.2)	261 (56.7)	
Stroke	Diagnosis	14 (1.6)	9 (1.9)	0.25 (0.618)
	None	879 (98.4)	456 (98.1)	
Hypertension	Diagnosis	569 (63.7)	314 (67.5)	1.95 (0.163)
	None	324 (36.3)	151 (32.5)	
CVD	Diagnosis	98 (11.0)	49 (10.5)	0.06 (0.806)
	None	795 (89.0)	416 (89.5)	
**Treatment**				
Diet	Yes	67 (7.5)	37 (8.0)	0.09 (0.765)
	No	826 (92.5)	428 (92.0)	
Oral Agent alone	Yes	756 (84.8)	399 (86.2)	0.43 (0.512)
	No	135 (15.2)	64 (13.8)	
Insulin	Yes	479 (53.8)	252 (54.4)	0.05 (0.815)
	No	412 (46.2)	211 (45.6)	
**Lifestyle**				
Smoker	Smokers	188 (21.7)	98 (21.7)	0.001(0.993)
	Ex/ non-smokers	680 (78.3)	354 (78.3)	
Exercise	Plan	272 (30.5)	135 (29.0)	0.30 (0.586)
	None	621 (69.5)	330 (71.0)	

*Test statistic represents t-tests for continuous, chi-test for categorical variables.
